# Non‐Volatile Resistive Switching in Nanoscaled Elemental Tellurium by Vapor Transport Deposition on Gold

**DOI:** 10.1002/advs.202406703

**Published:** 2024-10-01

**Authors:** Sara Ghomi, Christian Martella, Yoonseok Lee, Penny Hui‐Ping Chang, Paolo Targa, Andrea Serafini, Davide Codegoni, Chiara Massetti, Sepideh Gharedaghi, Alessio Lamperti, Carlo Grazianetti, Deji Akinwande, Alessandro Molle

**Affiliations:** ^1^ CNR IMM Unit of Agrate Brianza via C. Olivetti 2 Agrate Brianza 20864 Italy; ^2^ Dipartimento di Energia Politecnico di Milano via Ponzio 34/3 Milano 20133 Italy; ^3^ Microelectronics Research Center The University of Texas at Austin Austin Texas 78758 USA; ^4^ STMicroelectronics via C. Olivetti 2 Agrate Brianza 20864 Italy

**Keywords:** conductive AFM, memristors, resistive switching, tellurene, tellurium, 2D materials

## Abstract

Two‐dimensional (2D) materials are promising for resistive switching in neuromorphic and in‐memory computing, as their atomic thickness substantially improve the energetic budget of the device and circuits. However, many 2D resistive switching materials struggle with complex growth methods or limited scalability. 2D tellurium exhibits striking characteristics such as simplicity in chemistry, structure, and synthesis making it suitable for various applications. This study reports the first memristor design based on nanoscaled tellurium synthesized by vapor transport deposition (VTD) at a temperature as low as 100 °C fully compatible with back‐end‐of‐line processing. The resistive switching behavior of tellurium nanosheets is studied by conductive atomic force microscopy, providing valuable insights into its memristive functionality, supported by microscale device measurements. Selecting gold as the substrate material enhances the memristive behavior of nanoscaled tellurium in terms of reduced values of set voltage and energy consumption. In addition, formation of conductive paths leading to resistive switching behavior on the gold substrate is driven by gold‐tellurium interface reconfiguration during the VTD process as revealed by energy electron loss spectroscopy analysis. These findings reveal the potential of nanoscaled tellurium as a versatile and scalable material for neuromorphic computing and underscore the influential role of gold electrodes in enhancing its memristive performance.

## Graphical abstract

In this study, the resistive switching behavior of nanoscaled tellurium films using conductive atomic force microscopy is revealed, offering valuable insights into their memristive functionality. It is demonstrated that this memristive behavior is maintained in cross‐point devices and can be further enhanced by using gold as the substrate material, resulting in lower set voltages and reduced energy consumption.

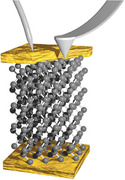

## 1 Introduction

The rapid development of information and communication technologies is reshaping the way we interact with our surrounding environment. Notably, the effective management of big data is becoming increasingly important to navigate the complexities of our ever‐changing environment. Despite the evident benefits derived from technological progress, significant challenges still exist and require advanced solutions in terms of newly engineered materials and operational paradigms. In this context, the demand for reducing power consumption has emerged as an urgent requirement for improving the efficiency and processing speed of electronic devices and systems. Traditional computing systems based on the von Neumann architecture that rely on the separation of memory and central processing unit,^[^ ^1^ ^]^ are incapable of fulfilling the performance demands of future technologies. As a response to this challenge, bio‐inspired approaches, drawing inspiration from the human brain, such as neuromorphic architectures, have gained popularity.^[^ ^2^ ^]^ Innovative designs emulating biological systems demonstrate the ability to simultaneously process large volumes of data while requiring minimal power consumption. In this context, resistive switching (RS) phenomena are emerging to implement the artificial synapses as a building block of a neuro‐inspired computational architecture, that is capable of storing and processing data in a single device.^[^ ^3^ ^]^ Briefly, the RS phenomena are characterized by cyclic changes in electrical resistivity between two distinct levels: high resistance state (HRS) and low resistance state (LRS).^[^ ^4^ ^]^ Non‐volatile RS enables the state of the system to be retained without requiring a constant power supply. The RS effect can be observed in a wide range of materials with strong implications for memory applications, such as resistive random‐access memories (RAM) or memristors. A memristor is a non‐volatile memory device that was mathematically predicted as the missing fourth circuit element (besides the resistor, capacitor, and inductor) by Chua^[^ ^5^ ^]^ in 1971 and experimentally demonstrated by Williams in Hewlett–Packard Laboratories in 2008.^[^ ^6^ ^]^

The physical origins of the RS phenomenon vary significantly depending on the materials employed and device architectures. They include conductive filament formation,^[^ ^7^ ^]^ crystallographic phase change,^[^ ^8^ ^]^ charge trapping/de‐trapping,^[^ ^9^ ^]^ ferroelectric and magnetic,^[^ ^10^, ^11^ ^]^ spin‐ and photo‐induced^[^ ^12^, ^13^ ^]^ switching mechanisms. Engineering the active material inside the memristive cell is a valuable strategy to substantially reduce the switching energy (and hence, the dissipated power) and time, no matter of the nature of the RS mechanism.

Various materials have traditionally demonstrated their implication in memristive devices including oxides.^[^ ^14^ ^]^ In particular, metal oxides are taken as constitutive building blocks for resistive switch RAM (so‐called ReRAM) where the memristive behavior is dictated by voltage‐induced effects like conductive filament formation, defect migration, ionic charge trapping, etc.^[^ ^15^, ^16^, ^17^ ^]^

In the ReRAMs framework, 2D materials have recently attracted interest in memristive applications as they can concomitantly offer superior performance and flexibility.^[^ ^18^, ^19^ ^]^ Reducing the material thickness down to the 2D limit, bears potential advantages in neuromorphic computing in terms of fast and low‐power switching, gate tunability, and mechanical robustness for flexible electronic applications.^[^ ^18^ ^]^ Recently, a wide range of 2D materials such as hexagonal boron nitride (h‐BN),^[^ ^20^, ^21^, ^22^, ^23^ ^]^ transition metal dichalcogenides (TMDs),^[^ ^24^ ^]^ and TMD‐based heterostructures^[^ ^25^, ^26^ ^]^ have been employed for the development of memristor devices. Among 2D materials, the use of monoelemental layers has emerged due to the simplification in the compositional chemistry and the higher homogeneity at the nanometre scale out of adventitious bonding defects (vacancies, antisite defects, etc.) in nanoscale compounds. These features are expected to hinder the performance variations observed in materials with a more complicated chemical composition typically used in RS schemes.^[^ ^27^ ^]^ Nonetheless, the consideration of elemental materials for memristor applications was limited to the case of black phosphorous (BP)/HfO_x_ bilayer as artificial synapsis,^[^ ^28^ ^]^ and no other alternative materials have been taken into account. One of the most recently discovered monoelemental 2D materials is tellurene, classified within the Xenes family,^[^ ^29^, ^30^ ^]^ and it is composed of covalently bonded tellurium atoms in helical chains connected by van der Waals forces.^[^ ^31^ ^]^ After Zhu et al. experimentally verified tellurene in 2017,^[^ ^32^ ^]^ numerous studies have been conducted renewing interest in tellurium.^[^ ^31^, ^33^, ^34^, ^35^, ^36^ ^]^ Tellurene exhibits a semiconducting character with a thickness‐dependent bandgap ranging from 0.3 eV in bulk up to 1.0 eV in the monolayer regime.^[^ ^37^ ^]^ Tellurene research is mostly focused on its potential applications in optoelectronics and electrical devices,^[^ ^38^ ^]^ such as chemical sensors,^[^ ^39^, ^40^ ^]^ and field effect transistors.^[^ ^34^, ^41^, ^42^, ^43^ ^]^ However, to date, the examination of the RS effect of synthetic tellurene at the nanoscale and the device level is relatively unexplored. As part of the investigation into the relatively new field of tellurium‐based RS devices, the influence of the substrate is revealed to be a significant factor in defining the overall operation of the device. The choice of substrate is pivotal in shaping how tellurium reconfigures within a memristor‐suited layout. Thus, the development of a synthesis method is crucial to achieve large‐scale ultra‐thin films of tellurium that may be applied to a variety of conductive substrates serving as bottom electrodes (BE).

In this work, we discuss the RS effect observed in ultra‐thin tellurium films deposited on a large scale by the vapor transport deposition (VTD) method at a sufficiently low temperature suitable for the effective exploitation of conductive materials as supporting substrates. Specifically, we use gold (Au) and tantalum nitride (TaN) as metallic substrates ensuring compatibility with the back‐end‐of‐line (BEOL) circuit fabrication process flow. To validate the RS behavior of tellurium at the local scale, we utilize the conductive atomic force microscopy (C‐AFM) technique, where the nanoprobe acts as a localized top electrode, eliminating the requirement for the microscale electrode deposition. Furthermore, we extend our investigation to the device level focusing on Au‐based substrates patterned by optical lithography to define the top and bottom electrodes. This study lays the groundwork for the utilization of the 2D form of tellurium directly deposited on metallic substrates for the investigation of memristive devices in technology‐compliant platforms readily transferable to edge‐computing systems.

## 2 Results and Discussion

### 2.1 Large‐Area VTD Growth of Ultra‐Thin Tellurium Films

**Figure 1:**
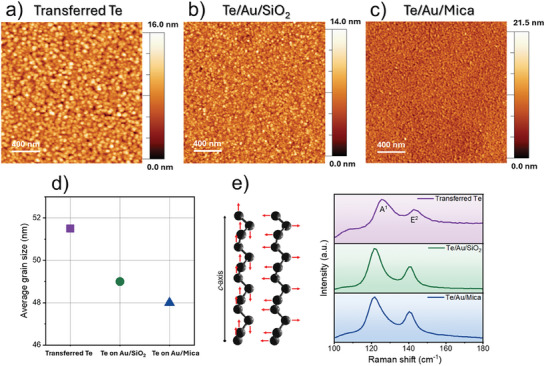
AFM topography characterization performed on 2 µm × 2 µm scan area of tellurium thin‐films a) transferred on Au/Mica substrate, b) directly grown on Au/SiO_2_ substrate and c) directly grown on Au/Mica substrate d) Comparison of average grain size calculated using cross‐correlation method e) Raman spectroscopy acquired on tellurium thin films transferred on Au/Mica substrate, directly grown on Au/SiO_2_ and Au/Mica substrates.

The ultra‐thin tellurium films were grown via the VTD method on a cm^2^ scale area at the growth temperature of 100 °C (Figure S1, Supporting Information). Two different approaches were considered for the substrate choice. First, tellurium was deposited on a SiO_2_/Si substrate (details in Figure S2, Supporting Information), followed by a transfer to a conductive supporting substrate. Second, direct growth on metallic substrates like Au/SiO_2_ and Au/Mica. The experimental results, including the transferred tellurium from the SiO_2_/Si substrate to the Au/Mica substrate, and the directly grown tellurium on Au/SiO_2_ and Au/Mica are summarized in **Figure** **1**. More details on the polymer‐assisted transfer methodology can be found in the Experimental Section and Supporting Information.

The tellurium growth demonstrated at a temperature as low as 100 °C^[^ ^29^ ^]^ further supports the choice of such a temperature also for gold substrates, the same used for the transferred tellurium, for the relevance that this metal has in many RS systems^[^ ^44^ ^]^ and to rule out any effect of polymer contamination during the transfer procedure. The direct growth on gold substrates represents a technological step toward the “transfer‐free” and “litho‐free” approaches to device manufacture with clean interfaces between the active material and the electrode, taking advantage from the intrinsic inert nature of gold against oxidation.^[^ ^45^ ^]^ Furthermore, gold not only provides a functional platform for memristive cell fabrication, but we also demonstrate that it plays a crucial role in boosting memristive performance. We anticipate that keeping the growth conditions under control is crucial for preventing the formation of AuTe_2_ as a result of the reaction between Au and Te. In particular, we found out that the temperature of the gold substrate should not exceed 350 °C.^[^ ^46^ ^]^

The high‐resolution AFM morphology images measured on 2 µm × 2 µm scan area of transferred tellurium, and directly grown tellurium on Au/SiO_2_ and Au/Mica substrates are represented in Figure 1a–c, respectively (with the thickness of 15 nm as shown in Figures S3 and S4, Supporting Information). Based on the cross‐correlation method, the topographies show the formation of continuous tellurium grains with typical grain sizes of 51.5 nm for transferred tellurium on Au and 49 and 48 nm for tellurium directly grown on Au/SiO_2_ and Au/Mica respectively (summarized in Figure 1d).

The Raman spectra of transferred tellurium on Au/Mica (Figure 1e, top panel) show two main peaks at 125.8 and 143.4 cm^−1^. These peaks are assigned to the A^1^ and E^2^ Raman active modes of the tellurium lattice in agreement with previous studies.^[^ ^31^, ^36^, ^47^ ^]^ The two modes correspond to the out‐of‐plane, characteristics of basal plane stretching along the *a*‐direction and in‐plane active mode due to asymmetric axial‐chain stretching along the *c*‐direction of the tellurium lattice (see Figure 1e).^[^ ^36^ ^]^ We stress that, along with the AFM characterization, Raman spectroscopy is routinely used to assess the quality of transferred films as well as 2D materials.^[^ ^48^ ^]^ Therefore, the evidence that the width and the relative intensity of the characteristic Raman peaks are comparable in the as‐grown and transferred material leads us to the conclusion that the polymer‐assisted transfer methodology has a negligible impact on the quality of the tellurium film placed onto the Au/Mica supporting substrate. A direct comparison of the spectra reported in Figure 1e reveals that the Raman modes of the tellurium grown on the Au/SiO_2_ substrate (middle panel) match with those detected on the equivalent deposition carried out on the Au/Mica substrate (bottom panel), see also SI and Figure S5 (Supporting Information). This is confirmed by the two peaks at 121.7 and 140.8 cm^−1^ associated with the A^1^ out‐of‐plane and E^2^ in‐plane vibrational modes of Te, which turn out to be slightly redshifted with respect to those of tellurium grown on SiO_2_/Si and then transferred to the gold substrate. The observed shift can be attributed to strain effects and/or substrate interactions after the transfer of the film.^[^ ^49^ ^]^

### 2.2 Local Resistive Switching Investigation of Tellurium Films using C‐AFM

**Figure 2:**
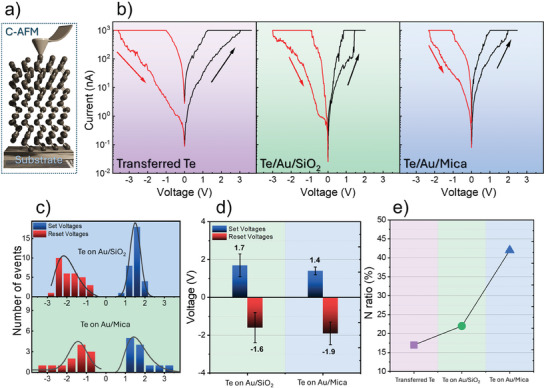
a) Schematic illustration of C‐AFM technique with a sharp tip acting as top electrode scanning over the tellurium films b) *I–V* characteristics obtained by C‐AFM performed on tellurium thin‐films transferred on Au/Mica, directly grown on Au/SiO_2_, and directly grown on Au/Mica substrate (from left to right) c) Comparison of a statistical distribution of set (blue) and reset (red) voltages d) Comparison of the average values and standard deviations of set and reset voltages e) Comparison of the calculated N ratio, a statistical parameter representing the ratio of the number of points with hysteretic RS behavior to the total number of acquired *I‐V* curves in different ultra‐thin tellurium films.

Employing nanoscale‐resolution electrical characterizations such as C‐AFM can enhance our understanding of the fundamental RS characteristics of ultra‐thin tellurium films. This approach allows for high‐resolution current mapping and local current–voltage (*I–V*) characterization prior to the macroscopic electrical investigation. We used C‐AFM to locally investigate the vertical electrical transport properties of the 2D tellurium films by recording the current between the conductive tip and the substrate in contact mode (see **Figure** **2**a) thus acquiring the topographic and current maps simultaneously. A more interesting picture of the electrical properties of the tellurium film is derived by the acquisition of point spectroscopy *I–V* curves on a statistical set of points in the AFM scanning maps. Figure 2b shows representative *I–V* curves obtained at specific points of the transferred and as‐grown tellurium films where a clear hysteretic feature can be observed by sweeping back the voltage ramp which is indicative of the RS behavior. In detail, in Figure 2b (left panel), the transferred tellurium film with a gold substrate as BE featured low currents corresponding to the HRS without an abrupt change up to ≈3.0 V (limited to prevent degradation of the AFM tip). Sweeping back the voltage, the material keeps to the LRS that persists until a negative voltage of −3.5 V is applied (again limited) thus resetting the LRS back to HRS. Hence the set and reset voltages can be assumed to be >3.0 and <−3.5 V, respectively.

For the tellurium grown on Au/SiO_2_ (Figure 2b center panel), as the positive voltage sweeps from 0 to 2 V, the set voltage amounts to 1.4 V where the transition from HRS to LRS occurs. With the negative bias, the resistance is switched back to the HRS at the reset voltage of −3.0 V. For the tellurium grown on Au/Mica (Figure 2b right panel) the set voltage is 1.9 V, and as the voltage decreases toward negative values, at the average reset voltage of −2.3 V it switches back to the HRS. The switching performance observed in tellurium directly grown on different gold substrates exhibits a bipolar RS behavior as well as the transferred one.

The set and reset voltage histogram distributions corresponding to all the hysteresis *I–V* measures are plotted and compared in Figure 2c (except for the transferred tellurium). The set voltage for tellurium grown on Au/SiO_2_ spans from 1.1 to 3.2 V and the reset voltage falls within the −3.3 up to −0.6 V range. For the tellurium grown on Au/Mica the set voltage value is between 0.8 to 2.0 V and the reset voltage value is between −2.7 to −1. V. As the latter configuration looks more promising, the double‐logarithmic *I–V* curves for the set and reset processes of ultra‐thin tellurium films grown on Au/Mica are depicted in Figure S6 (Supporting Information).

The comparison of the observed voltage variations indicates how the RS behavior changes under various substrate conditions. More importantly, the behavior of the *I–V* curves directly proves that the deposited tellurium films behave as a switch in selected points only, thereby suggesting an intrinsic origin of the mechanism in ultra‐thin tellurium films being present in both samples. This observation echoes what has already been observed in switching devices based on 20 nm thick Te cells, where the physical mechanism for the resistive switch consists of a transition from crystalline to liquid phases of the Te films induced by the Joule heating effect.^[^ ^50^ ^]^

In Figure 2d,e, the C‐AFM measurements were statistically analyzed to compare the average values and variations of both set and reset voltages of the tellurium films directly grown on gold substrates. Considering that surface Au atoms are expected to be highly mobile undergrowth, it is interesting to assess if the Au substrate in the direct growth scheme plays an extra role in triggering the RS effect more efficiently than in the other cases in point. A more thorough comparison was also conducted using tellurium films directly grown on a different metallic substrate such as TaN to further explore the role of gold in boosting RS behavior. The results of *I–V* characteristics obtained by C‐AFM on tellurium directly grown on TaN substrate are reported in Figure S7 (Supporting Information). Notably, the average set voltage values and their variations for tellurium directly grown on Au/SiO_2_ and Au/Mica substrates are calculated to be 1.7 ± 0.6 V and 1.4 ± 0.2 V respectively which are lower than those of transferred tellurium 2.2 ± 0.9 V and of the tellurium grown on TaN substrate 4.7 ± 0.8 V. Furthermore, the average values of the reset voltage and their variations for the tellurium directly grown on Au/SiO_2_ and Au/Mica substrates are calculated to be −1.6 ± 0.8 V and −1.9 ± 0.6 V while the corresponding values for the transferred tellurium and tellurium grown on TaN are –3.7 ± 1.5 V and −5.6 ± 1.1 V. On the other hand, Figure 2e summarizes the calculated statistical parameter N which is defined as the ratio of the number of points with hysteresis RS behavior with respect to the total number of acquired *I–V* curves. The overall comparison elucidates that the tellurium films directly grown on Au substrates considerably increase the number of RS points, whereas the transferred tellurium sample and tellurium grown on TaN exhibit much lower percentages of RS points in the probed area with the calculated N ratio of 18% and 7% respectively (see Figure 2e; Figure S7, Supporting Information).

The lower average set and reset voltages, their variations, and the higher yield of directly grown tellurium on Au substrates suggest the favorable characteristics of the direct growth method in terms of the reduction of voltage and energy consumption. Such a comparison better clarifies that the best RS performances, in terms of reduction of the voltages (and hence of power consumption), are observed in the samples obtained by the direct deposition of tellurium on Au. This fact points out the primary role of the Au substrate in boosting the intrinsic RS behavior of the tellurium film. As a matter of fact, Au has been used as conductive material for the BE in memristor devices based on MoS_2_ (atomristor) as an active layer.^[^ ^24^ ^]^ In such an atomristor configuration, the RS behavior has been attributed to the gold ion migration from the BE through the atomic vacancies located in the MoS_2_ active layer.^[^ ^51^ ^]^ We notice that the local nature of the C‐AFM measurements is typically called out to attribute the origin of the RS behavior to a filamentary conduction mechanism as shown in Figure S6 (Supporting Information). The lower average set and reset voltages, their variations, and the higher yield of directly grown tellurium on Au substrates suggest the favorable characteristics of the direct growth method in terms of the reduction of voltage and energy consumption. Such a comparison better clarifies that the best RS performances, in terms of reduction of the voltages (and hence of power consumption), are observed in the samples obtained by the direct deposition of tellurium on Au. This fact points out the primary role of the Au substrate in boosting the intrinsic RS behavior of the tellurium film. As a matter of fact, Au has been used as conductive material for the BE in memristor devices based on MoS_2_ (atomristor) as an active layer.^[^ ^24^ ^]^ In such an atomristor configuration, the RS behavior has been attributed to the gold ion migration from the BE through the atomic vacancies located in the MoS_2_ active layer.^[^ ^51^ ^]^ We notice that the local nature of the C‐AFM measurements is typically called out to attribute the origin of the RS behavior to a filamentary conduction mechanism as shown in Figure S6 (Supporting Information).

### 2.3 Resistive Switching Mechanism and Role of Gold

**Figure 3:**
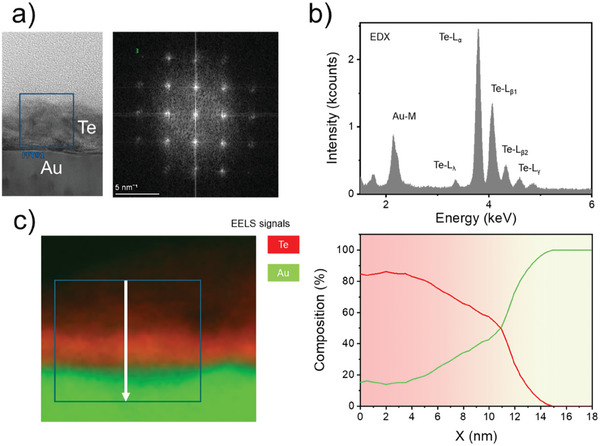
a) cross‐sectional STEM images showing the deposited Te film on the Au substrate along with the FFT electron diffraction pattern of the lattice corresponding to the region of the blue box. b) EDXS spectrum showing the detected electronic shells of Au and Te atoms in the same box of a). c) EELS map and percent composition along the path in figure (white arrow). The spatial distribution of Au and Te shows that the two energy‐loss signals overlap at the interface over a scale of several nanometers.

To have a closer insight into the role of the gold substrate in the direct tellurium growth and thus unveil the origin of the RS mechanism, cross‐sectional images of the tellurium film deposited on gold substrates were acquired using an aberration‐corrected scanning transmission electron microscopy (STEM). The deposited tellurium film is fully crystalline as assessed by calculating the electron diffraction pattern of the lattice by fast Fourier transform (FFT) corresponding to the rectangular box in **Figure** **3**a (see also Figure S8, Supporting Information for more details). While the Te layer appears homogeneous in the STEM micrograph (Figure 3a left), the corresponding energy‐dispersive x‐ray spectroscopy (EDX) (Figure 3b) reveals that both elements are present in the deposited material. We emphasize that, while the most prominent peaks in the EDX spectrum correspond to the L‐subshell of Te, a significant presence of Au is identified by the M‐subshell of the gold peaked at ≈2.2 keV. This fact demonstrates that even the low thermal budget of the Te deposition at 100 °C is sufficient to activate the diffusion mechanism of the gold atoms from the substrate into the deposited material. Since the EDX analysis typically suffers from poor spatial resolution, we also display in Figure 3c the electron energy‐loss spectroscopy (EELS) graphs acquired in the rectangular selection of the STEM image. The EELS offers very high spatial resolution and enables us to draw out a detailed elemental analysis of the deposited material.^[^ ^52^ ^]^ Notably, we find that Te and Au's signals overlap on a scale of several nanometers at the interface of the two materials, which is clearly displayed in the spectrum extracted along the path indicated in the energy‐loss map (see white arrow in Figure 3c). In this overlapping region, the percent composition of the elements can be semi‐quantitatively assessed by using the Hartree–Slater scattering cross‐sections of the M shells of the elements implemented in the analytical software, which are Te ≈ 60% ± 9% and Au ≈ 40% ± 6%.^[^ ^52^ ^]^

### 2.4 Investigation of Resistive Switching Capability in Te Film Cross‐point Memristors

**Figure 4:**
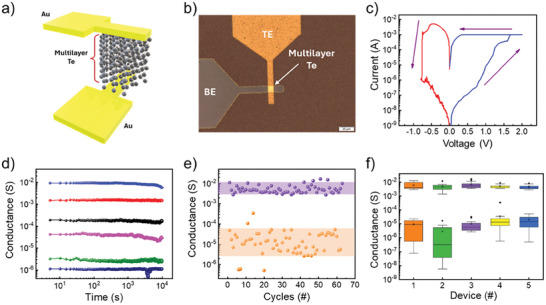
a) Schematic of the cross‐point memristor based on vertical Au/tellurium/Au device array. b) Optical microscope image of the device. c) *I−V* curve of the device resistive switching. d) Multi‐level states retention as long‐term memory analysis. Reliability analysis: e) DC switching endurance test over 60 cycles and f) device‐to‐device uniformity with box plot.

The interplay of the local memristive behavior and the low thermal budget of the growth make the tellurium films readily integrated into a device architecture. As a device demonstrator, we fabricated a cross‐point memristor layout with a 5 × 5 µm^2^ junction area defined by top and bottom Au electrodes (see Experimental Section and Supporting Information for fabrication details). The schematic of the tellurium device is reported in **Figure** **4**a, while Figure 4b shows the optical microscope image of the crossbar structures (Figure S9, Supporting Information). The RS active media is composed of an ultra‐thin film of tellurium (15 nm thickness) grown by VTD. After fabrication, the electrical characterization of the devices is performed in a DC probe station (refer to the Experimental Section for details).

As shown in Figure 4c, the device displays a bipolar switching voltage sweep and a maximum on/off ratio up to 137000. Figure 4d presents the retention characteristics in multilevel states by switching with the compliance current. From 1 µS (HRS) to 10 mS (LRS), each 6 state conductance level indicates data‐retention capability greater than 10^4^ s, which is indicative of a nonvolatile and stable performance. The endurance characteristic of the device operation is presented over 60 continuous cycles in Figure 4e. We report in Supporting Information the detailed *I–V* switching for the endurance test, Figures S10–S11 (Supporting Information). Quantitatively, the set voltage sweep step size is 5 mV, with return points at 0.78 V for Figure S11a (Supporting Information). The reset voltage sweep step size is 2 mV and the average return points are at −0.45 V for Figure S11b (Supporting Information).

We notice that the cross‐point architecture enables the utilization of RS behavior from the device unit level to a scalable circuit stage with high on‐chip device density. To ensure reliability, it is crucial to study the devices fabricated at various spatial positions within the sample. We conducted this characterization by assessing device‐to‐device and cycle‐to‐cycle uniformity in devices spaced 0.5 to 1 cm apart.

The conductance distribution of LRS and HRS for device‐to‐device reliability is presented in Figure 4f for 5 devices with more than 15 cycles. We observed that in each device, the resistance (LRS and HRS) states could be clearly distinguished, and they exhibit comparable values within the statistical distribution of the measurements. As a final comment on the device characteristics, we interestingly noticed that the cross‐point devices show not only the ambipolar switching behavior described in Figure 4 but also a unipolar switching behavior as described in Figure S12 (Supporting Information). This observation necessitates a more thorough investigation into the physical origins of the resistive switching mechanism in ultra‐thin tellurium films.

## 3 Conclusion

We investigate the potential of large‐area ultra‐thin tellurium films grown via the VTD method at low temperatures for non‐volatile RS behavior in memristive device configurations. Memristors based on nanoscaled tellurium films demonstrate the RS switching behavior both at the local scale as probed by C‐AFM and at the device level in a micro‐scale cross‐point layout. In the former case, we show that C‐AFM‐based memristors demonstrate an ambipolar behavior which is promising for low‐power operation when nanoscaled tellurium is grown directly on gold substrates. Similarly, in the latter case, implementing top micro‐scaled contacts in a cross‐point architecture, we demonstrated the bipolar switching characteristics of the nanoscaled tellurium films with the endurance of over 60 cycles using gold as both top and bottom electrodes. By combining these different methodologies, we conclude that using the gold substrate as a bottom electrode plays a pivotal role in boosting the performance of tellurium‐based memristors. This finding opens a wide room to investigate parametric optimization of the so‐developed tellurium‐based memristors and to engineer them in flexible circuitry taking benefit from the extraction of the nanoscaled tellurium‐based membrane. This is fully compatible with the back‐end‐of‐line processing which is essential for providing a perspective for the Internet of Things applications. Furthermore, the exploitation of tellurium film heterostructures with other 2D materials can be explored to develop innovative memristor designs with enhanced characteristics.

## 4 Experimental Section

### Materials Growth

Vapor Transport Deposition on two different gold substrates was used for the experiments: i) 50 nm‐thick gold film was deposited onto SiO_2_ (50 nm)/Si substrate employing e‐beam evaporation. ii) commercial Au (111) single crystal with nominal thickness ≈300 nm on mica substrate. The deposition reactor consists of a double furnace system equipped with a quartz tube reactor of 2′′ diameter. Tellurium (Te) powder (40 mg: 99.997%, Sigma–Aldrich, Darmstadt, Germany) was used as a precursor. The tellurium powder was placed in a ceramic boat in the center of the upstream furnace, and the gold substrate was cut in 3 × 1 cm^2^ dimensions, kept on a ceramic boat (face up), and positioned 18 cm away from the Te precursor reference. The temperatures of 440 °C set for the upstream and 100 °C set for the downstream, with a 100 sccm Ar/H_2_ flux (H_2_ 4% volume) as a carrier gas that was flowing for 30 min growth time.

### Transfer Methods

The transfer process of centimeter‐scale tellurium was done via the wet transfer method by Hydrofluoric acid (HF). First, a layer of Poly (methyl methacrylate) (PMMA) was spin‐coated with 1500 rpm spinning speed onto the Te/SiO_2_/Si stack acting as a support layer and then baked at 80 °C for 15 min. Then, the PMMA/Te/SiO_2_/Si stack was floated in HF solution until complete detachment from the underneath SiO_2_/Si substrate. Once the PMMA/Te was detached, the floating stack was fished to the target Au/Mica substrate and baked at 90 °C for 1 min. The remaining PMMA was removed by acetone completing the transfer process.

### Sample Characterization—Atomic Force Microscopy (AFM)

The morphology of the samples was investigated in tapping mode using commercial AFM (Bruker Dimension Edge). Topographies were acquired in tapping mode using ultra‐sharp silicon tips (TESPA‐V2 Bruker radius of curvature 7 nm nominal frequency 320 kHz) Statistical parameters of the surface morphology, such as root‐mean‐square (RMS) Roughness, were derived by means of freely available software (WSxM, Gwyddion).

### Sample Characterization—Conductive AFM (C‐AFM)

The commercial AFM (Bruker Dimension Edge) equipped with a TUNA electrometer with a 1 pA to 1 µA current range was used to characterize the electrical properties of the samples at the local scale. A conductive diamond‐coated tip (CDT‐CONTR, nanosensors, with a radius of curvature between 100 and 200 nm) was used to scan over the surface of the sample in ambient conditions.

### Sample Characterization—Raman Spectroscopy

The vibrational properties of the deposited sample were verified by Raman spectroscopy in z‐backscattering geometry using a Renishaw spectrometer (In‐Via) equipped with a solid‐state laser source of excitation wavelength 514 nm/2.41 eV. The laser source was coupled with an optical microscope and objective with numerical aperture = 0.75 and magnification 50×. The laser power on the sample was kept below 5 mW to avoid sample damage.

### Sample Characterization—Electrical Testing

The cross‐point structure Te memristor was measured to verify electrical characteristics using the Keithley 4200‐SCS semiconductor parameter ultrafast module and a 4225‐RPM pulse signal unit. All measurements were carried out by controlling the top electrode bias and fixing the bottom electrode as ground.

### Transmission Electron Microscopy (TEM), EDX and EELS

The samples were extensively investigated utilizing scanning transmission electron microscopy (STEM) related techniques such as dark‐field STEM (DF‐STEM) for the microstructural characterization, while the chemical properties were investigated by STEM‐EELS and STEM EDX. DF‐STEM, EDX, and EELS analyses were carried out on electron‐transparent lamella obtained using focused ion beam (FIB) thinning of cross‐section TEM lamellae. The lamellae preparation was performed using a Thermofisher Helios G5UX FIB. In all the cases, particular care was taken to limit the heating and ballistic effects of ion irradiation on the samples during the final ion milling steps. In particular, a new advanced approach has been applied, reducing currents and energies progressively from a value of 30 keV to a value of low keV in order to avoid material amorphization and damage. The STEM images were performed with a Thermofisher Themis Z G3 aberration‐corrected scanning transmission electron microscope equipped with an electron gun monochromator operating at 200 kV acceleration voltage. To limit the electron beam damage, all the STEM images, EDX, and EELS maps were acquired with a low beam current (0.5 nA). The EELS experiments were performed with the post‐column Quantum Gatan imaging filter operating with an energy resolution of 1 eV/channel. The Te and Au elemental maps were obtained with a step size of 8 Å, and the data were processed using the Gatan GMS Digital Micrograph 3.23 software.

### Sample Processing

The isolated cross‐point structures were realized on a 300 nm SiO_2_/Si substrate. Both the electrodes (top and bottom) were patterned through optical lithography by using the Tabletop Maskless Aligner µMLA from Heidelberg. After defining the pattern of the bottom electrodes, SiO_2_ was partially etched using a buffered oxide etchant (BOE) solution, consisting of hydrofluoric acid (HF) and ammonium fluoride (NH_4_F) with a volume ratio of HF: NH_4_F = 1:7. After the etching of 130 nm of SiO_2_ in 1 min and 30 sec, the electrodes were realized through e‐beam evaporation of 5 nm of Ti and 50 nm of Au carried out using the Auto 304 system evaporator from Edward, equipped with a quartz microbalance. After the lift‐off process and the growth of 15 nm of tellurium, the top electrodes were patterned and realized by using the previous techniques and depositing 100 nm of Au via e‐beam deposition.

## Conflict of Interest

The authors declare no conflict of interest.

## Author Contributions

All authors have given approval to the final version of the manuscript.

## Supporting information



Supporting Information

## Data Availability

The data that support the findings of this study are available from the corresponding author upon reasonable request.
