# Supplementary material for: Non‐Volatile Resistive Switching in Nanoscaled Elemental Tellurium by Vapor Transport Deposition on Gold
Source: Adv Sci (Weinh). 2024 Oct 1;12(1):2406703. doi: 10.1002/advs.202406703 (PMC11714190; doi:10.1002/advs.202406703)
Supplement: Supplementary file 1 — Supporting Information [file ADVS-12-2406703-s001.docx]

**Supporting Information**

Non-volatile resistive switching in nanoscaled elemental tellurium by vapor transport deposition on gold

S. Ghomi^1,2^, C. Martella^*1^, Y. Lee^3^, P. H.-P. Chang^3^, P. Targa^4^, A. Serafini^4^, D. Codegoni^4^, C. Massetti^1^, S. Gharedaghi^1^, A. Lamperti^1^, C. Grazianetti^1^, D. Akinwande ^*3^ and A. Molle^*1^

^1^ CNR IMM, Unit of Agrate Brianza, via C. Olivetti 2, Agrate Brianza 20864, Italy

^2^ Dipartimento di Energia, Politecnico di Milano, via Ponzio 34/3, Milano 20133, Italy

^3^ Microelectronics Research Center, The University of Texas at Austin, Austin, Texas 78758, United States

^4^ STMicroelectronics, via C. Olivetti 2, Agrate Brianza 20864, Italy

Corresponding Author

* christian.martella@cnr.it * deji@ece.utexas.edu * alessandro.molle@cnr.it

**Growth conditions for achieving large-area ultra-thin tellurium films via VTD**

***
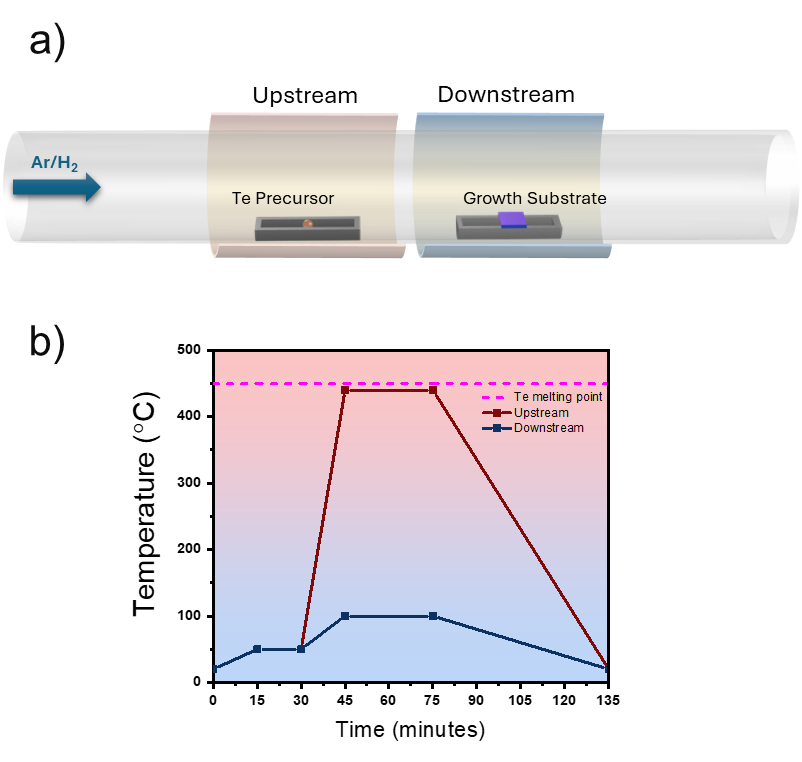
***

**Figure S1.** a) Schematic representation of hot-wall tube double furnaces systems with tellurium powder precursor and substrate boats b) Temperature ramp applied to the upstream and downstream furnaces

**Raman spectroscopy and AFM on ultra-thin tellurium films grown on SiO_2_/Si substrate before transfer to Au/Mica substrate**

The Raman spectra were acquired on the tellurium films grown on SiO_2_/Si, before transfer (solid line), and after transfer on Au (111)/Mica (dashed line) process. The peak positions remain approximately unchanged, while the intensity of the peaks arises after the transfer due to the background coming from the gold substrate.


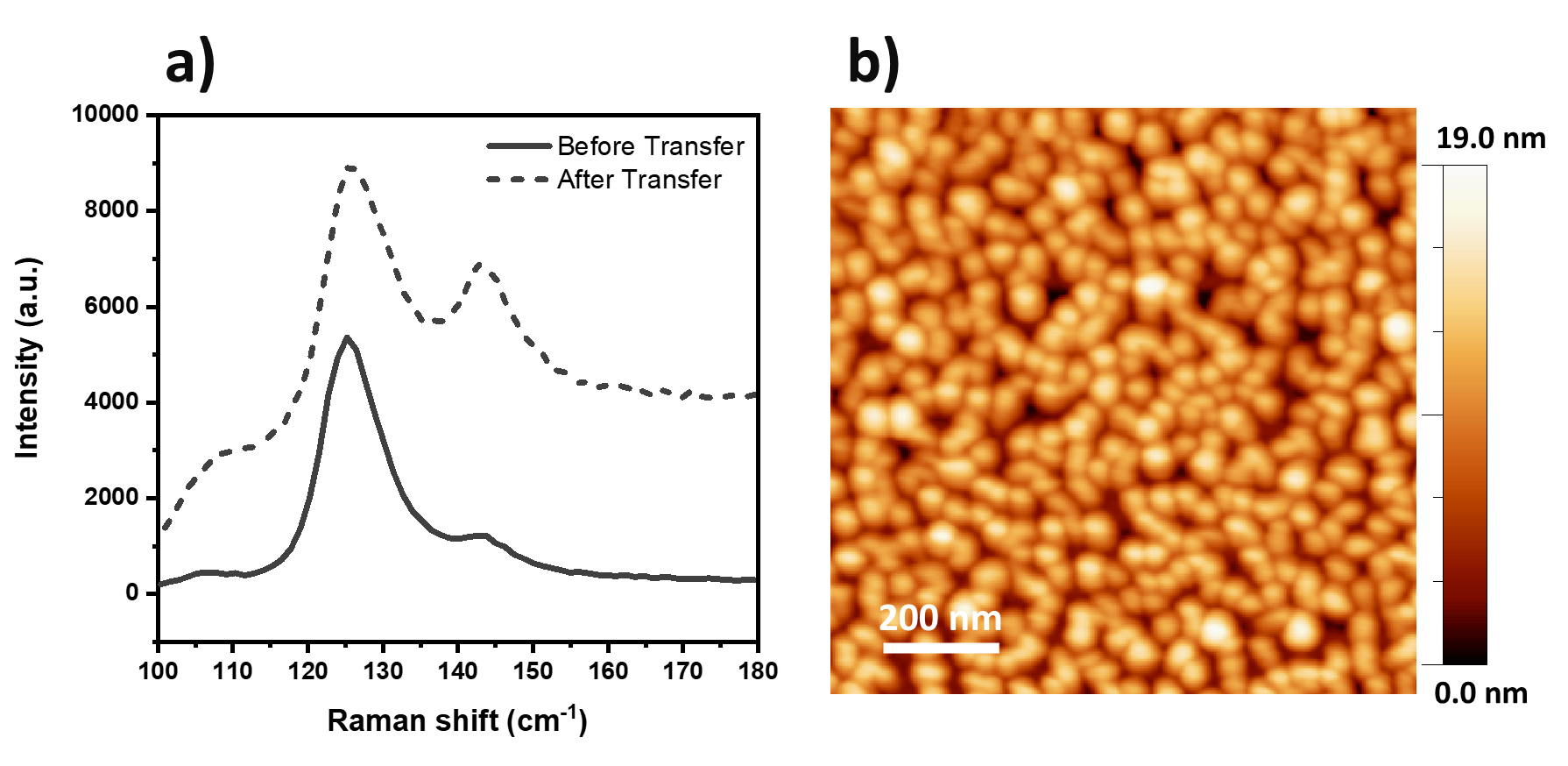
**Figure S2.** a) Raman spectra acquired on the ultra-thin tellurium film grown on SiO_2_/Si substrate before (solid line) and after (dashed line) transfer to the Au substrate b) AFM topography image of the ultra-thin tellurium film grown on SiO_2_/Si substrate before transfer.

**Thickness measurements of ultra-thin tellurium films with AFM**

**
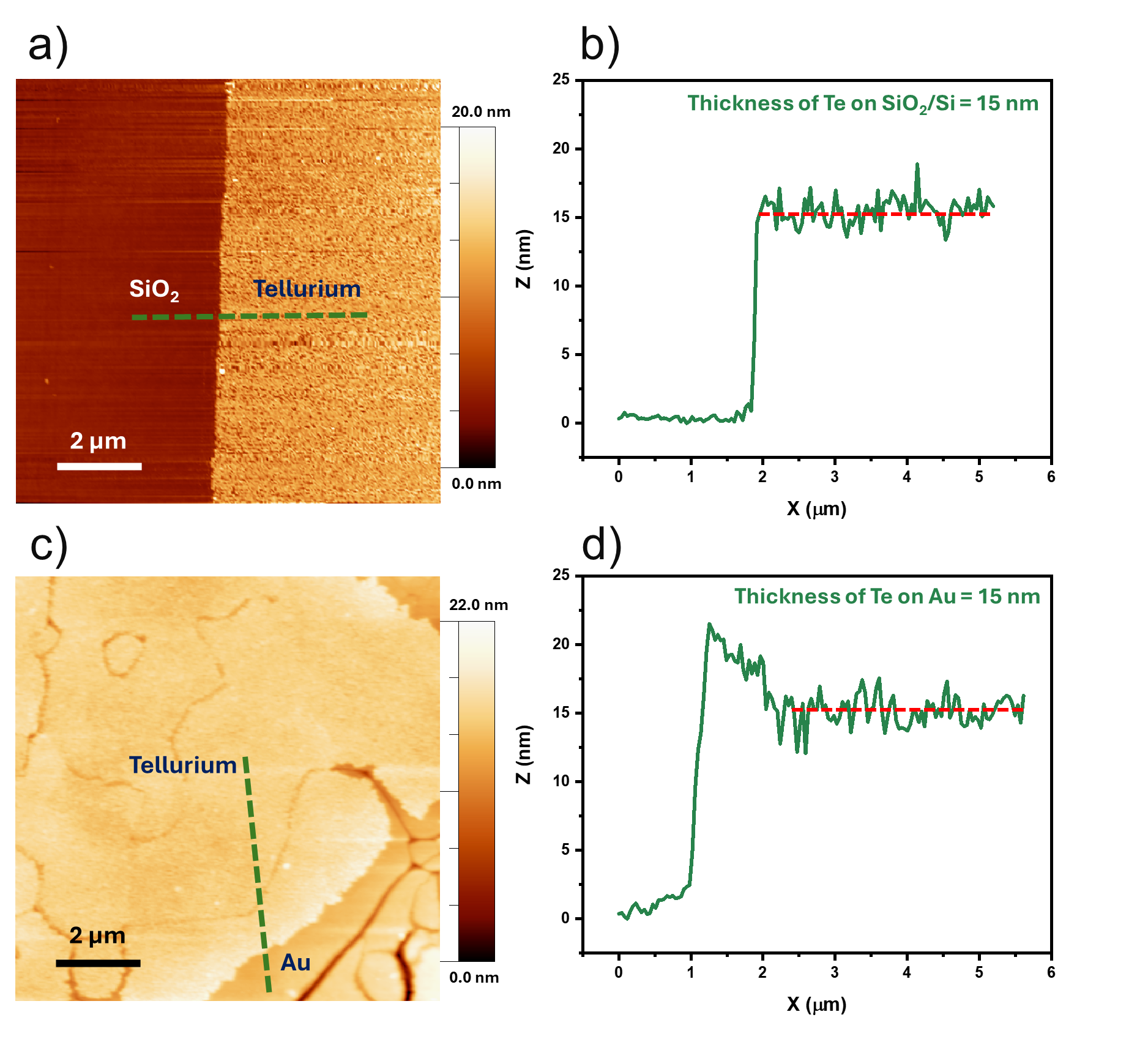
**

**Figure S3**. a) AFM topography image of the scratched, deposited tellurium on SiO_2_/Si (before transfer) b) height profile along the depicted dashed green line, indicating a thickness of 15 nm. c) AFM topography image of the etched, directly VTD grown tellurium on gold substrate d) height profile along the depicted dashed green line, indicating the thickness of 15 nm.

**AFM morphologies of ultra-thin tellurium films at different spatial positions**

We also investigated the uniformity of deposited Te through AFM morphology analysis across different scan areas over the grown samples on gold substrates: 1) transferred Te on gold, 2) Te directly grown on Au/SiO2, 3) Te directly grown on Au/mica. The results are shown in Figure S4. In the three cases, the morphology of the deposited tellurium is compatible with that of a compact film constituted by small (nanometer scale size) polycrystalline grains. The statistical analysis of the surface parameters reveals that, despite the different nature of the supporting gold substrates (polycrystalline vs single crystal), the deposited material has comparable root-mean-squared (RMS) roughness values in the range 1-2 nm. The uniformity of the deposition is further supported by comparing the morphologies and surface parameters acquired at three different spatial positions on the sample surface separated by a distance of 0.3 cm.


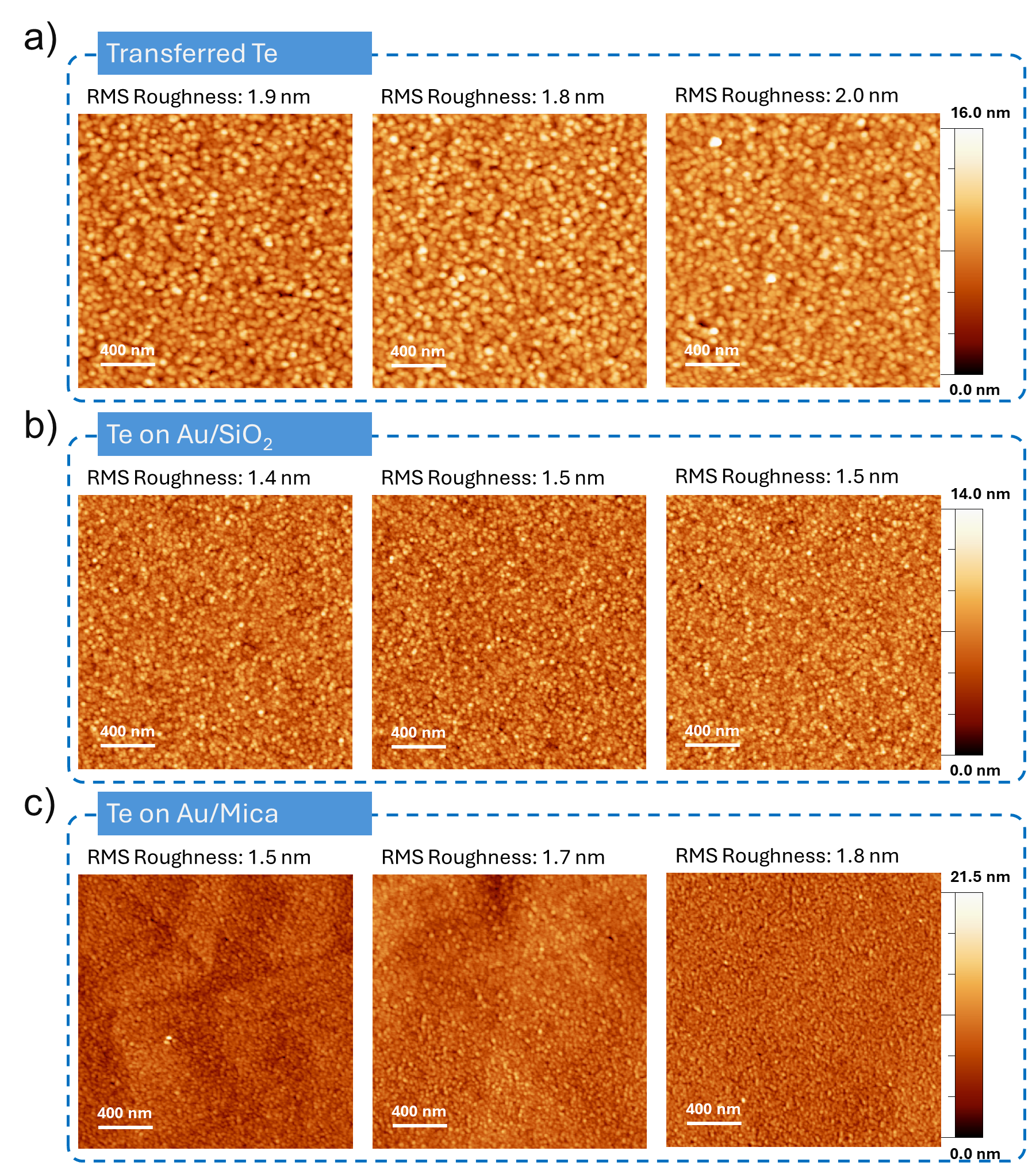


**Figure S4.** AFM topography characterization performed on 2 μm × 2 μm scan area, acquired at three different spatial positions on the sample surface separated by a distance of 0.3 cm of tellurium thin-films a) transferred from SiO_2_/Si to gold substrate b) deposited on Au/SiO_2_/Si and c) deposited on Au/Mica substrates.

**Uniformity of the deposited ultra-thin tellurium films**

Te film is uniformly deposited over the area of 1 cm × 1 cm which can be supported by measuring the Raman spectra at 9 different points arranged in a 3 × 3 grid. To illustrate this picture, we firstly assess the mean value of the A^1^ and E^2^ frequency peak positions and the relative spectral dispersion inferred as the standard deviations of the peak values compared to the mean value. Figure S5 a) shows that, for both Te films deposited on Au/SiO_2_/Si and Au/Mica substrates, the variation of the peak positions of the Raman modes is less than 0.5% compared to the mean value. Additionally, we compared in Figure S5 b) the full-width-half-maximum (FWHM) of the Raman modes for both the samples. We observe that the analysis of the FWHM of the Raman modes is commonly used to assess the degree of order or disorder in materials, which is related to their crystallinity, such as crystalline domain size and defectivity. Quantitatively, the average FWHM values are 8-9 cm⁻¹ for the A^1^ peak and 7-8 cm⁻¹ for the E^2^ peak of the samples. The FWHM values reflect the polycrystalline nature of the samples, characterized by grains with limited crystalline domain size (on the order of tens of nanometers, according to AFM investigation). Despite the limited variation compared to the average value (with standard deviation indicating variation of the FWHM within 7%), this demonstrates the overall good uniformity of the samples in terms of crystalline properties. Finally, we focus our attention on the spatial Raman intensity distribution for the A^1^ peak. We created a distribution map showing the Raman intensity of A^1^ peak based on the coverage area. The map clearly indicates that both Te films deposited on Au/SiO_2_/Si and Au/Mica substrates exhibit minimal variation of the peak intensity (of the order of 10%) across the growth area, confirming highly uniform deposition, Figure S5 c).


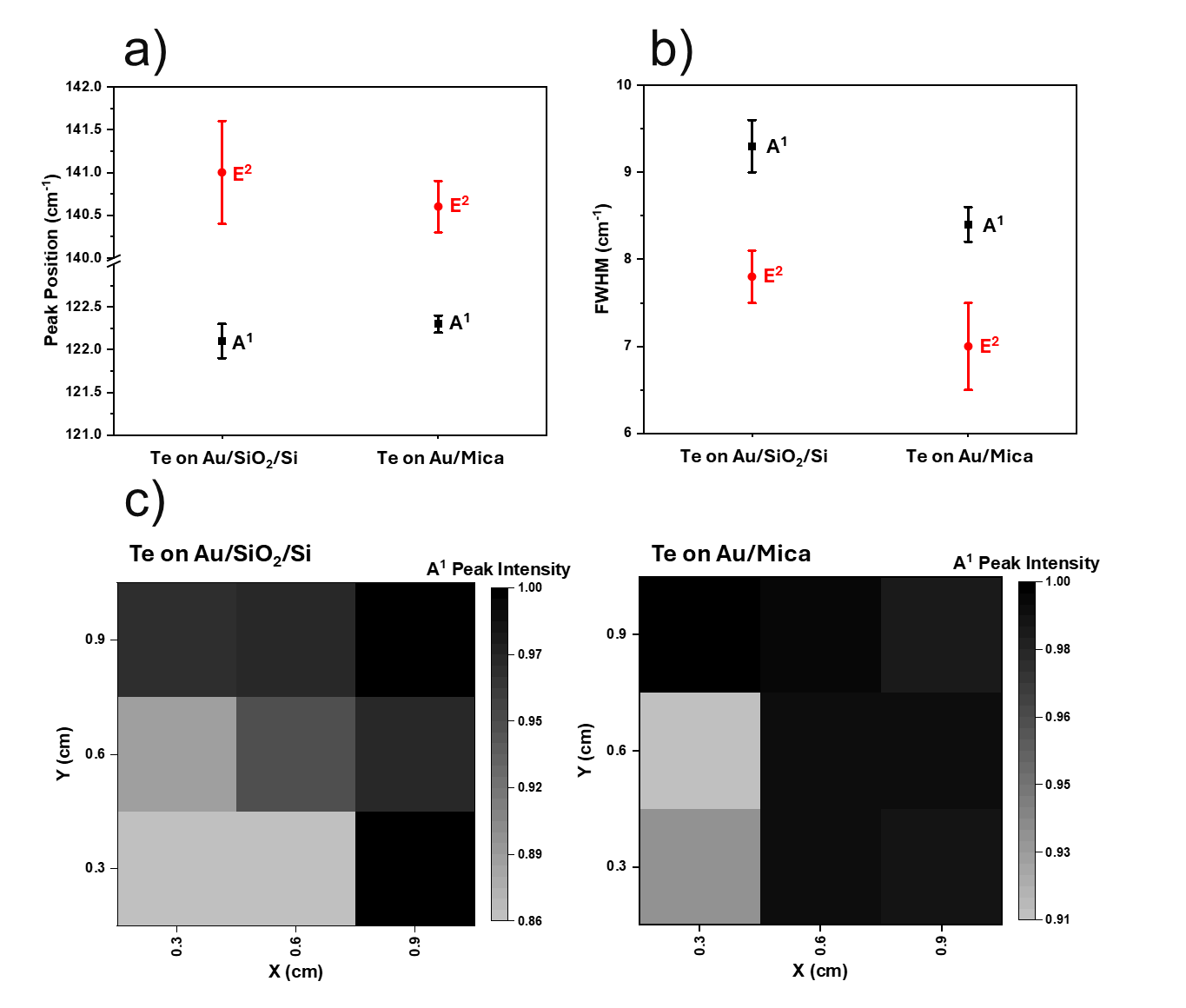


**Figure S5.** a) comparison of the mean value and standard deviations of the A1 and E2 frequency peak positions for the VTD grown tellurium on Au/SiO2/Si and Au/Mica substrates b) comparison of the FWHM of the A1 and E2 Raman modes c) comparison of the spatial Raman intensity distribution for the A^1^ peak.

**Revealing the conduction mechanism at the local scale through log(|I|) vs. log(|V|) plot for tellurium films directly grown on a gold substrate**

According to the literature [1], the driving forces behind the formation of conductive filaments and RS behavior in thin films include the complex interplay between various factors. One contributor is the presence of vacancies introduced naturally in the films. Simultaneously, the diffusion of gold atoms within the film matrix, potentially facilitated by the existence of vacancies, adds another contributor to the mechanism. A voltage sweep can trigger the gold atoms within the film due to their tendency to be absorbed into the vacancies, facilitated by the negative adsorption energy observed for the adsorption of gold into a vacancy site in various 2D materials. [2] Increasing the voltage can lead to the accumulation of gold atoms and complete the formation of conductive paths, leading to the SET process. Then, sweeping the negative voltage initiates the removal of gold atoms from defects causing the RESET process and restoring the resistance state.

To further investigate the RS mechanism, the *I-V* characteristics of the tellurium films on Au (111)/Mica substrates during set and reset processes are plotted in a double-logarithmic axis (**Figures S6**). Analyzing the slope of the linear fit at different segments of the double-logarithmic plot provides insights into the underlying conduction mechanism. During the set process, the *I-V* curve in the LRS has a slope of ≈ 1 indicating the current and voltage follow Ohm’s law (I ≈ V) which suggests the conductive filament formation. [3] The HRS's initial slope shows the linear correlation associated with the ohmic conduction mechanism. As the voltage gradually increases, the slope shows a quadratic relation indicating the transition to a space charge limit conduction mechanism (SCLC). Subsequently, the slope undergoes an abrupt jump reaching 6.8, indicating a shift from trap-unfilled to trap-filled SCLC mechanism. The different conductive characteristics in HRS and LRS highlight the charge transport in the LRS is characterized by localized behavior, aligning with the conductive filament formation mechanism. [3] A similar process occurs during the reset process (**Figure S6**).


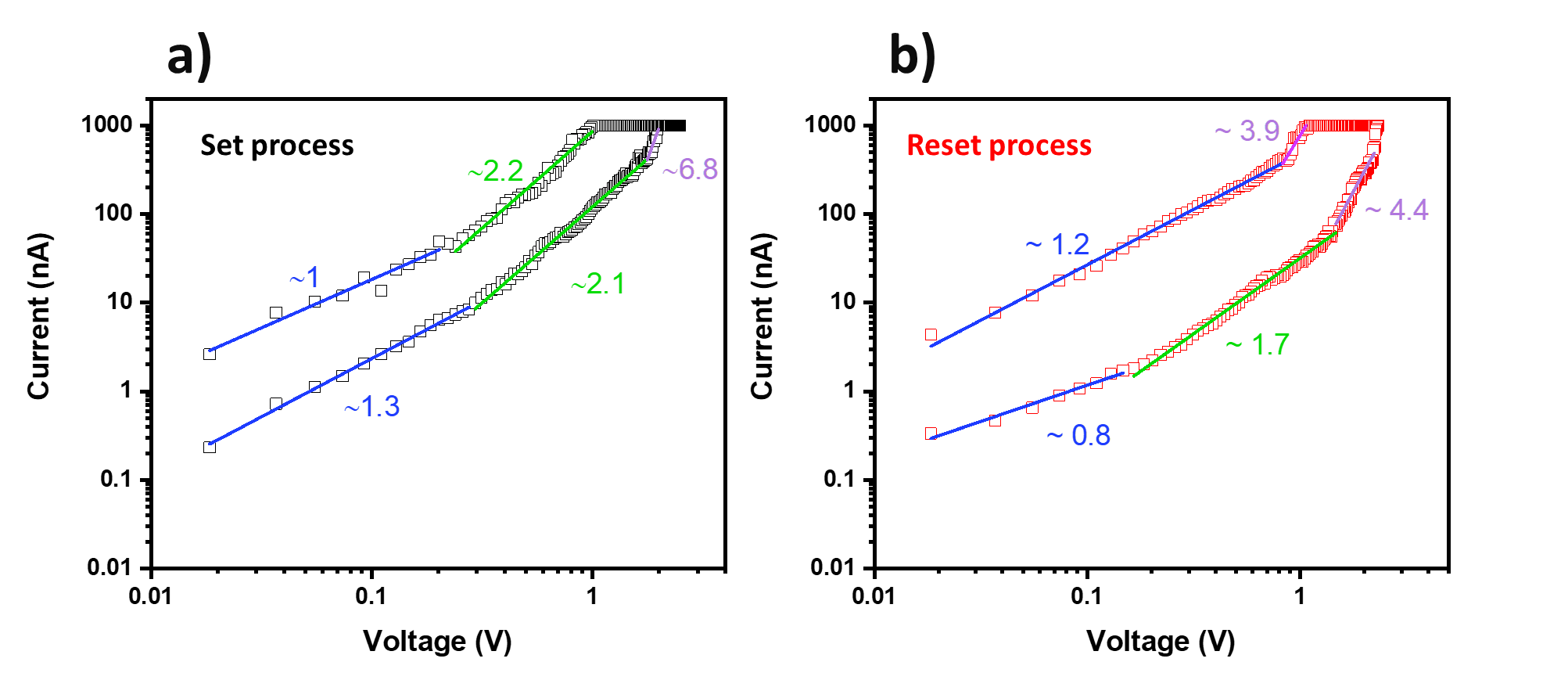
 **Figure S6.** Double-logarithmic plots of the *I-V* characteristics of ultra-thin tellurium films grown on Au (111)/Mica in a) positive bias region (set process) and b) negative bias region (reset process).

**Tellurium films on TaN substrate: Growth results and I-V characteristics**


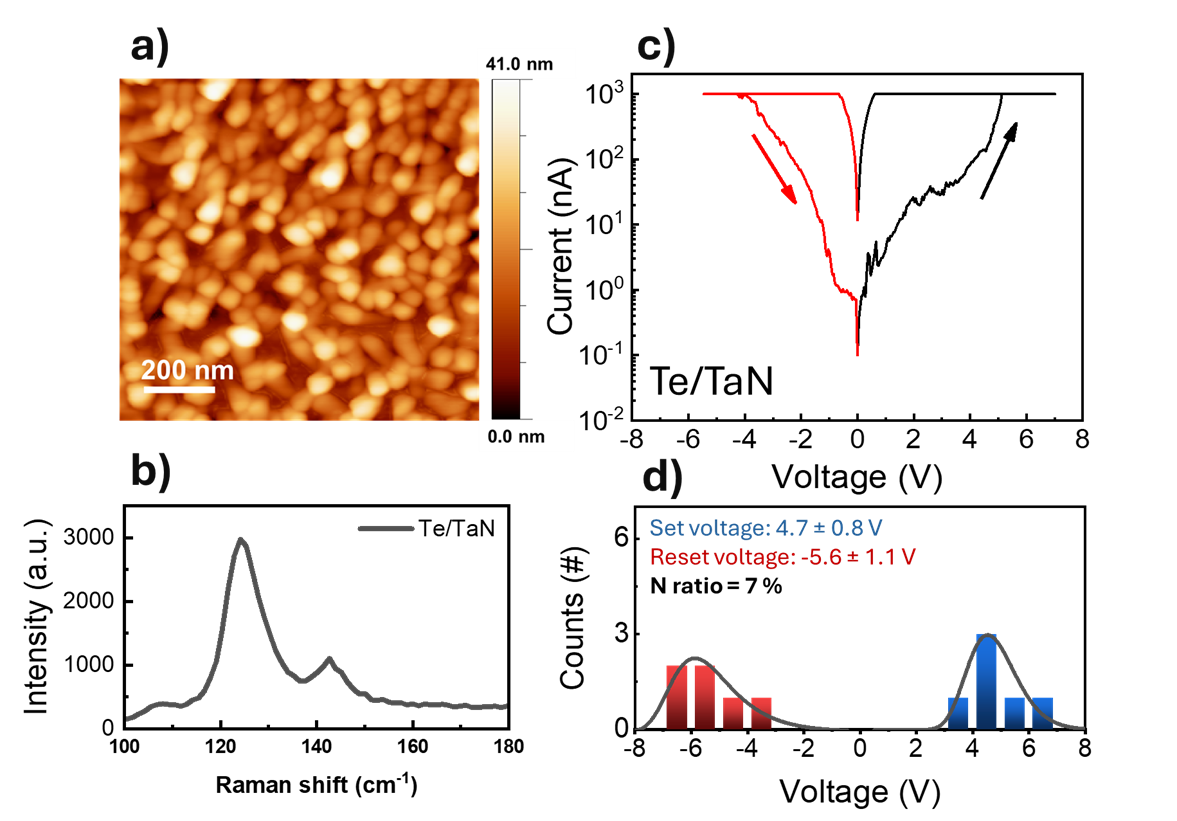


**Figure S7.** a) AFM topography image. b) Raman spectra. c) Representative I-V characteristics acquired by c-AFM technique. d) Distribution of set (blue) and reset (red) voltages for ultra-thin tellurium films grown on TaN substrate.

**TEM and XRD characterization of ultra-thin tellurium directly grown on gold substrates**

In Figure S8 b, we have calculated the spacing of the different crystallographic planes inferred from the fast-Fourier transformed (FFT) pattern related to selection evidenced by the blue square in Figure S8 a. From this analysis, we have determined the following lattice spacing distances: d= 0.33 nm; 0.22 nm. These values are in good quantitative agreement with the distances of the (101) and (102) planes, respectively, as reported by M.S.Kim et al. Adv. Mater. 2018, 30, 1702701. This attribution is further confirmed by the X-ray diffraction (XRD) pattern reported in Figure S8 c)


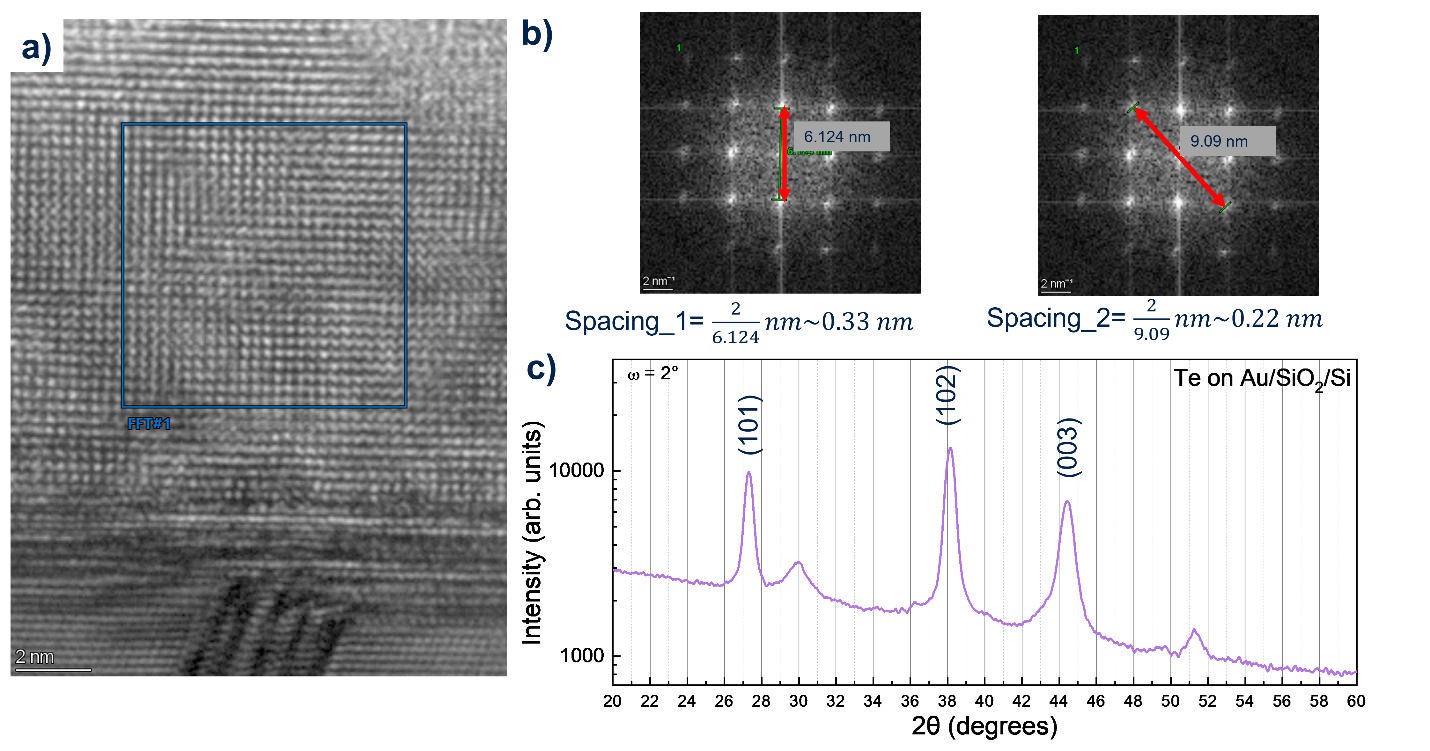


**Figure S8.** a) Large scale cross-sectional TEM image and b) FFT of the blue square box of a.c) XRD pattern of the Tellurium film deposited on gold

**Memristor device cross-point fabrication process based on ultra-thin tellurium films deposited on Au/SiO_2_/Si *
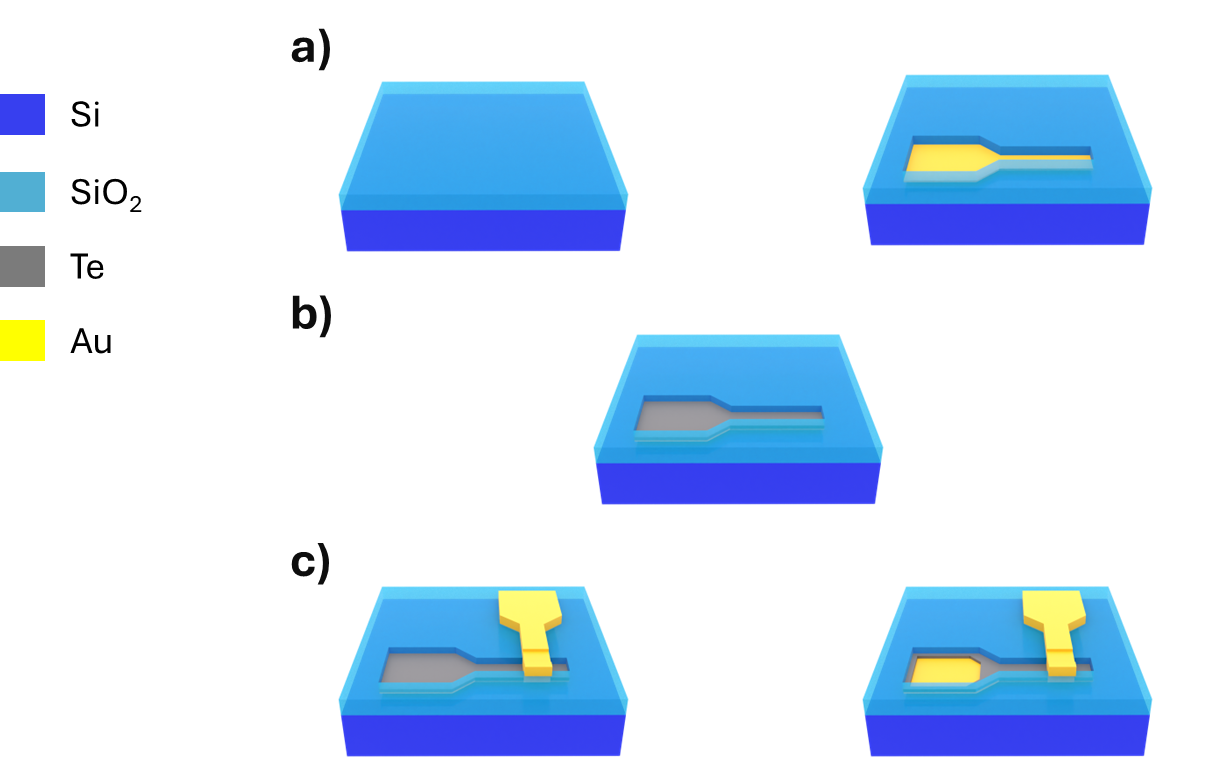
***

**Figure S9.** Schematic illustration of the fabrication process of the cross-point memristor device. a) Bottom electrode pattern definition on SiO_2_ (300 nm) / Si substrate by an optical maskless lithography technique, a partial SiO_2_ etch of 130 nm, deposition of 5 nm and 50 nm Ti and Au by e-beam evaporation followed by lift-off. b) Direct vapor transport deposition of ultra-thin tellurium films. c) Top electrode pattern definition by an optical maskless lithography technique, deposition of 5 nm and 100 nm Ti and Au by e-beam evaporation followed by lift-off.

**Detailed I−V switching for the endurance test and a quantitative comparison of the distribution of set and reset voltages in cross-point memristor devices**


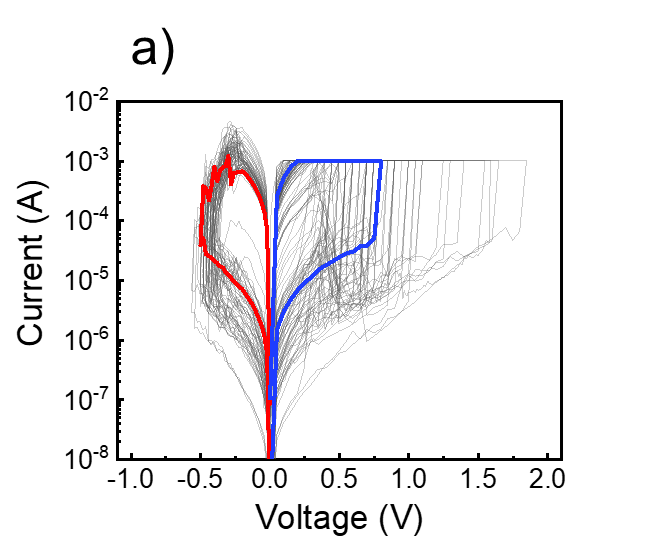


**Figure S10.** I−V curve of 60 resistive switching cycles during DC endurance test.


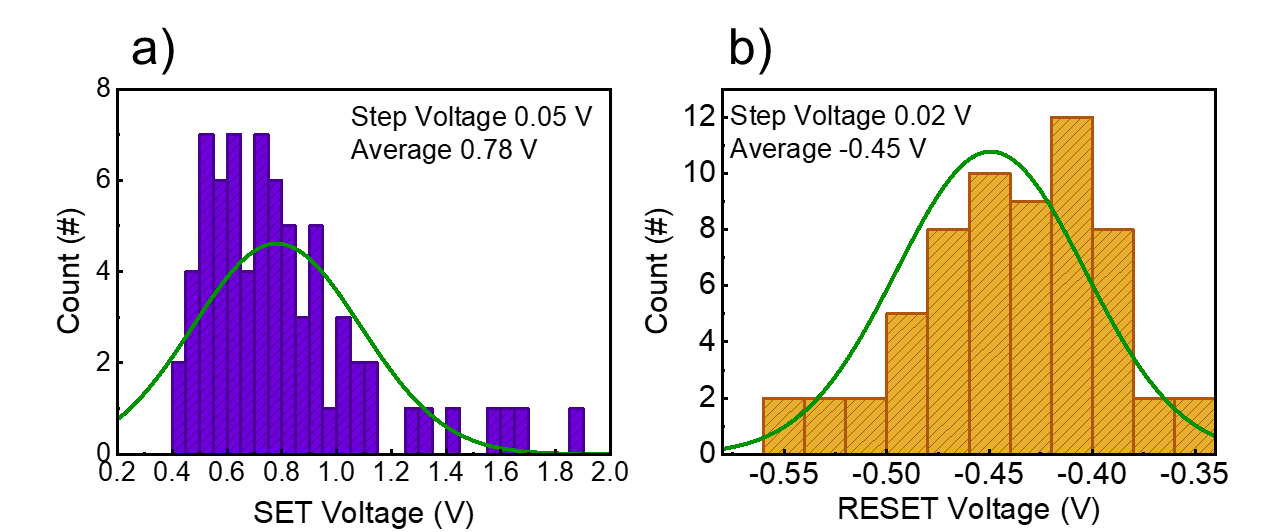


**Figure S11.** a) Distribution of set voltage during DC endurance test. b) Distribution of reset voltage during DC endurance test.

**Investigation of the unipolar I-V characteristics in cross-point memristors**


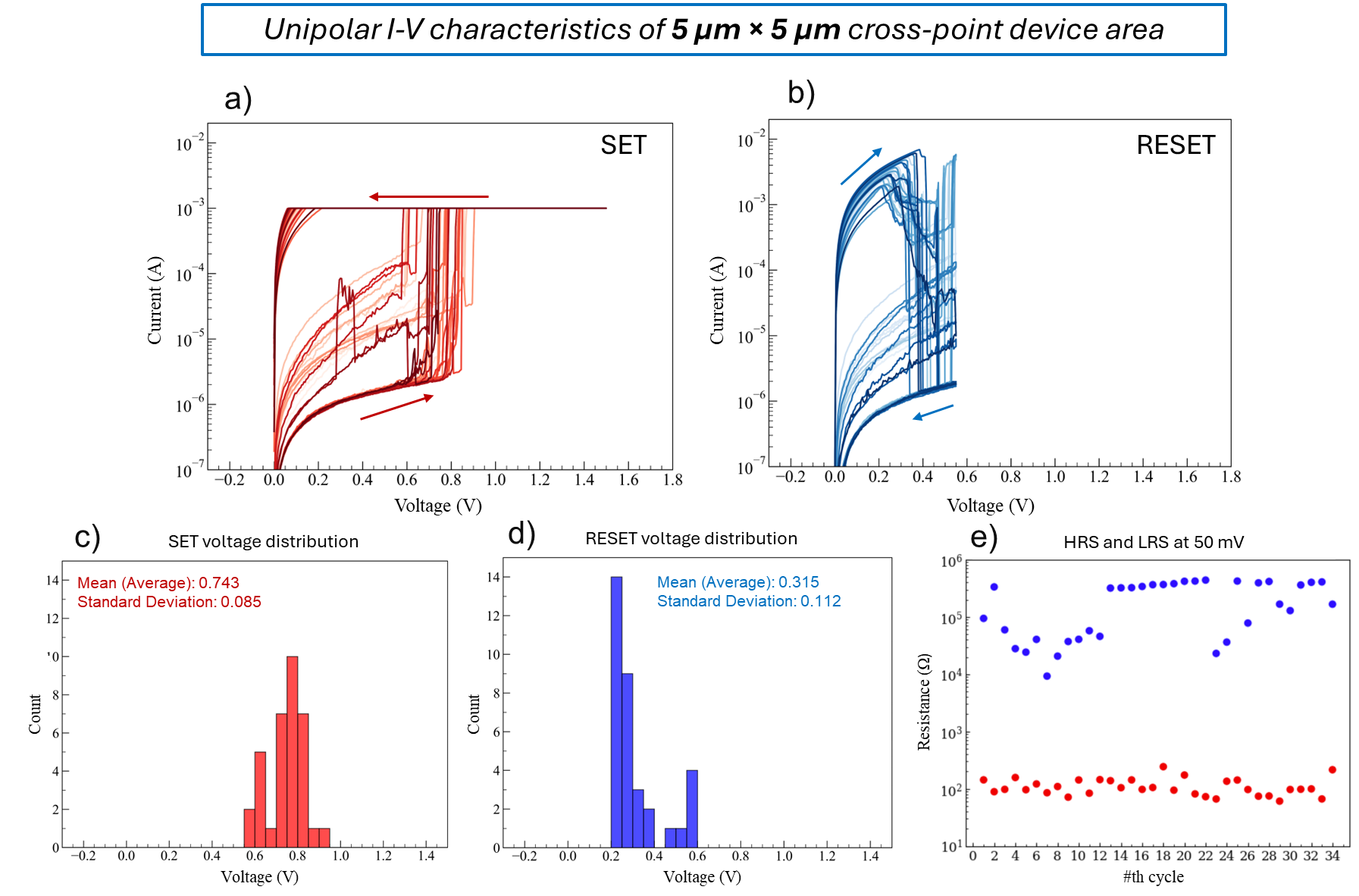


**Figure S12**. Unipolar switching of Te cross-point memristor with 5 µm × 5 µm area over 34 cycles a) Set cycles. (b) Reset cycles. (c) Histogram of the set voltages. (d) Histogram of the reset voltages. (e) HRS (blue) and LRS (red) values for each unipolar switching cycle.

**References**

1. Huang, Y., Gu, Y., Mohan, S., Dolocan, A., Ignacio, N.D., Kutagulla, S., Matthews, K., Londoño-Calderon, A., Chang, Y.-F., Chen, Y.-C., Warner, J.H., Pettes, M.T., Lee, J.C., and Akinwande, D. Reliability Improvement and Effective Switching Layer Model of Thin-Film MoS2 Memristors. *Advanced Functional Materials*, **n/a** (n/a), 2214250.

2. Ge, R., Wu, X., Liang, L., Hus, S.M., Gu, Y., Okogbue, E., Chou, H., Shi, J., Zhang, Y., Banerjee, S.K., Jung, Y., Lee, J.C., and Akinwande, D. (2021) A Library of Atomically Thin 2D Materials Featuring the Conductive-Point Resistive Switching Phenomenon. *Advanced Materials*, **33** (7), 2007792.

3. Lin, W., Zhuang, P., Akinwande, D., Zhang, X.-A., and Cai, W. (2019) Oxygen-assisted synthesis of hBN films for resistive random access memories. *Applied Physics Letters*, **115** (7), 073101.
